# Effects of Photoperiod and Light Quality on Germination and Growth of *Camellia sinensis* ‘HuangKui’

**DOI:** 10.3390/plants13131782

**Published:** 2024-06-27

**Authors:** Gan Hu, Xingchen Li, Junlong Yang, Qingqing Yuan, Shijun Yang, Wenjun Fu, Xianchen Zhang, Yeyun Li, Zhougao Shen, Jiayue Jiang

**Affiliations:** State Key Laboratory of Tea Plant Biology and Utilization, Anhui Agricultural University, Hefei 230036, China; 15798095067@stu.ahau.edu.cn (G.H.); 22720024@stu.ahau.edu.cn (X.L.); 23720469@stu.ahau.edu.cn (J.Y.); yuaqingqing@stu.ahau.cn (Q.Y.); yangshijun@stu.ahau.cn (S.Y.); fwj21720021@stu.ahau.edu.cn (W.F.); zhangxianchen360@163.com (X.Z.); liyeyun360@163.com (Y.L.); kjcszg@ahau.edu.cn (Z.S.)

**Keywords:** photoperiod, light quality, tea plants, germination

## Abstract

Light, as a critical environmental factor, plays a pivotal role in photosynthesis, ultimately influencing the timing of bud flush in tea plants. However, the synergistic effects of different photoperiods and light qualities on the timing of bud flush in the albino tea cultivar ‘HuangKui’ (later germination variety) remain unknown. Thus, the objective of this study was to investigate the effects of different photoperiods (12L/12D, 14L/10D, 16L/8D, and 18L/6D, where L = the number of daylight hours and D = the number of hours of darkness) and ratios of red (R) to blue (B) light (R/B 1:1, R/B 1:2, R/B 1:3, and R/B 2:1) on the germination and growth of the albino tea variety ‘HuangKui’. In our study, we examined how different photoperiods and red light and blue light affected tea germination and growth by investigating the timing of bud flush, photosynthesis, chlorophyll content, and growth indicators. First, our study showed that ‘HuangKui’ germinated 4 days, 2 days, and 1 day earlier under the 16L/8D photoperiod at the one bud and one leaf period compared with plants cultivated under the 12L/12D, 14L/10D, and 18L/6D photoperiods under light simulating the solar spectrum. Also, the growth of ‘HuangKui’ was maximumly promoted under the 16L/8D photoperiod treatment. Additionally, the earliest germination of ‘HuangKui’ was observed for the 16L/8D photoperiod under the R/B 2:1 (red/blue) treatment compared with the other treatments. Moreover, the greatest plant height, length of the new shoots, and new leaf areas were detected in the albino tea variety ‘HuangKui’ under R/B 2:1. Moreover, the contents of auxin (indole acetic acid, IAA) and trans-zeatin (tZ) under R/B 2:1 were significantly higher than those under the R/B 1:1 and control treatments with the 16L/8D photoperiod. Additionally, the auxin-related expression levels of *CsIAA13*, *CsGH3.1*, *CsAUX1*, and *CsARF2* under the R/B 2:1 treatment were significantly higher than those in the control. The expression of *CsARR-B*, a positive regulator of cytokinin-related genes, was significantly higher under the R/B 2:1 treatment than under the control treatment, while the opposite result was found for the expression of the negative regulator *CsARR-A*. Therefore, the R/B 2:1 treatment with the 16L/8D photoperiod was an appropriate means of timing the bud flush for the albino tea variety ‘HuangKui’, which may be related to IAA or tZ signal transduction. In conclusion, our research offers a novel lighting strategy that promotes the germination and growth of albino tea cultivars.

## 1. Introduction

The tea plant (*Camellia sinensis* (L.) O. Kuntze), a perennial woody plant, originated in southwestern China and is now extensively grown all over the world. Its fresh leaves can be used to make popular non-alcoholic beverages [1]. As a significant economic crop, the germination time of tea plants is an important agronomic trait, directly impacting tea production and local tea economic development. ‘HuangKui’ is one of the most representative albino tea cultivars [2]. Due to its limited chloroplast development and insufficient chlorophyll accumulation, the tender shoots exhibit a golden yellow color in early spring [3]. Therefore, the amino acid and theanine contents in ‘HuangKui’ are significantly higher than those in the green variety, resulting in its unique flavor. However, ‘HuangKui’ is a late-bud break tea cultivar [4], and thus, exploring the germination-promoting mechanisms in albino tea plants is an urgent task in the tea industry.

An increasing amount of evidence has shown that the exogenous application of plant growth regulators can promote tea growth. Recently, auxin (indole acetic acid, IAA), gibberellic acid (GA_3_), cytokinins (CKs), and brassinolide (BL) were shown to promote the germination of tea plant shoots effectively [5,6,7,8]. Di et al. [9] reported that an exogenous supply of melatonin (MT), which upregulates leucine-rich repeat (LRR) receptor-like serine/threonine-protein kinase and transcription factors (*CsMYB6*, *CsMYB7*, *CsWRKY40*, and *CsWRKY75*), promotes the growth of tea plants. Also, Lin et al. [10] found that the germination density of spring tea (Fudingdahao variety) increased from 8.58% to 15.24% with the foliar application of silicon fertilizer in early spring. In addition, the foliar application of magnesium promotes tea plant growth by enhancing glutamine synthetase-mediated nitrogen assimilation [11].

Additionally, the molecular biological approach for germination research in tea plants has achieved definite progress. *CsARP1*, *CsGLP1*, *CsARF1*, *CsPIN3*, *CsAUX1*, and *CsPILS2* have all been found to be closely related to dormancy lifting and tea shoot germination in tea plants [12]. Combined with quantitative trait loci (QTL) mapping, Tan et al. [13] found that two major QTLs (qSPI3 and qSPI4) play an important role in the spring germination of tea plants. Seven genes (three uncharacterized protein genes, two peroxidase genes, one uridine S’-monophosphate synthase gene, and one calmodulin-binding protein gene) affecting the timing of bud flush in tea plants were identified [14].

However, the above methods are time-consuming and have a low efficiency. Thus, it is very important to adopt appropriate measures to promote spring bud flush. With the continuous progress of technology, a novel lighting technology, LEDs (light-emitting diodes), with high efficiency, stability, and controllability, has been widely used in crop sprouting. For example, 50 μmol m^−2^ s^−1^ with a 12 h d^−1^ photoperiod is an optimal light condition for the shoot growth of ginseng (*Panax ginseng*) [15]. Chen et al. [16] reported that greater sprout length was observed under 16-h red and blue light conditions. In addition, higher levels of fresh weight, shoot height, and root length were detected in Snapdragon (*Antirrhinum majus* L.) under 50% blue and 50% red treatment compared with natural daylight treatment [17]. Similar results showed that the greatest leaf area and germination rate in arugula (*Eruca vesicaria* ssp. *Sativa*) were detected under a 24-h photoperiod with blue–red–far-red lighting [18]. The above experiments confirmed that the appropriate combination of LEDs can significantly enhance plant germination rates. However, the effect of red and blue light on germination in tea plants remains unknown.

In this study, different photoperiods and light qualities were provided to examine the germination and growth of ‘HuangKui’ (an albino tea variety). In addition, the contents and expression levels of hormone-related genes were detected to explore the underlying mechanism.

## 2. Results

### 2.1. Tea Plant’s Phenological Periods and Growth

**Figure 1:**
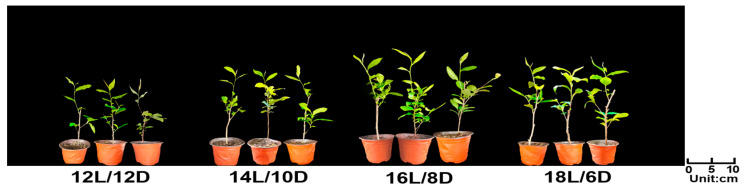
The effect of different photoperiod treatments on the growth of tea plants.

As shown in Figure 1, the best growth of tea plants was observed under the 16L/8D treatment (in all cases, the number refers to the number of hours, and L and D represent daylight hours and hours of darkness, respectively). The leaves of ‘HuangKui’ began to sprout at 8 days under the 16L/8D and 18L/6D treatments, while the leaves sprouted at 9 or 10 days under the 14L/10D and 12L/12D treatments. Additionally, the number of days until the appearance of one bud and one leaf, one bud and two leaves, and one bud and three leaves are shown in Table 1.

### 2.2. Leaf Photosynthetic Performance under Different Photoperiod Treatments

**Figure 2:**
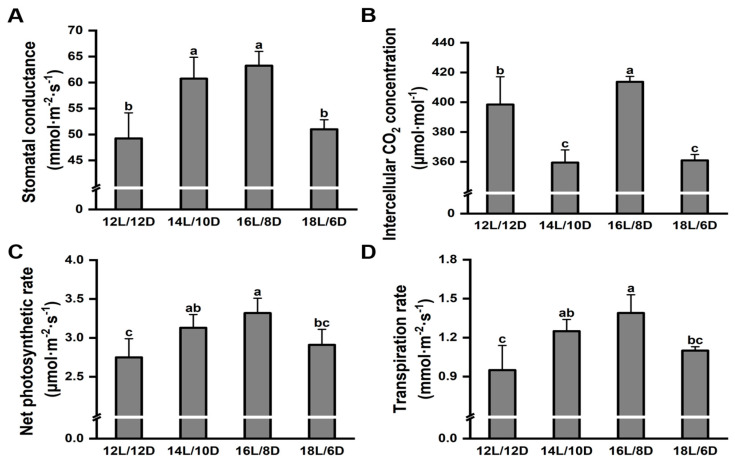
Effect of different photoperiod treatments on photosynthetic parameters of ‘Huangkui’. Effect of different photoperiod treatments on stomatal conductance (**A**), intercellular CO_2_ concentration (**B**), net photosynthetic rate (**C**), and transpiration rate (**D**) of ‘Huangkui’; Note: Different lowercase letters on the top of the bar chart indicate significant differences at the level of *p* < 0.05.

To further illustrate the effect of different photoperiod treatments on the growth of tea plants, the photosynthetic parameters were measured on the fully expanded second leaves of each treatment. Consistent with the above results, the highest levels of stomatal conductance (Figure 2A) and intercellular CO_2_ concentration (Figure 2B) were detected under the 16L/8D treatment. The photosynthesis of ‘HuangKui’ was significantly higher by 20.73% under the 16L/8D treatment compared with that under the 12L/12D treatment (Figure 2C). Also, similar results were found for the transpiration rate (Figure 2D).

### 2.3. Leaf Chlorophyll Content under Different Photoperiod Treatments

The chlorophyll level is an important component related to photosynthesis in plants. As shown in Table 2, the highest contents of chlorophyll A and chlorophyll B were detected under the 16L/8D photoperiod, being significantly higher than those under the 12L/12D and 14L/10D photoperiods.

### 2.4. Growth of Tea Plants under Different Photoperiod Treatments

**Figure 3:**
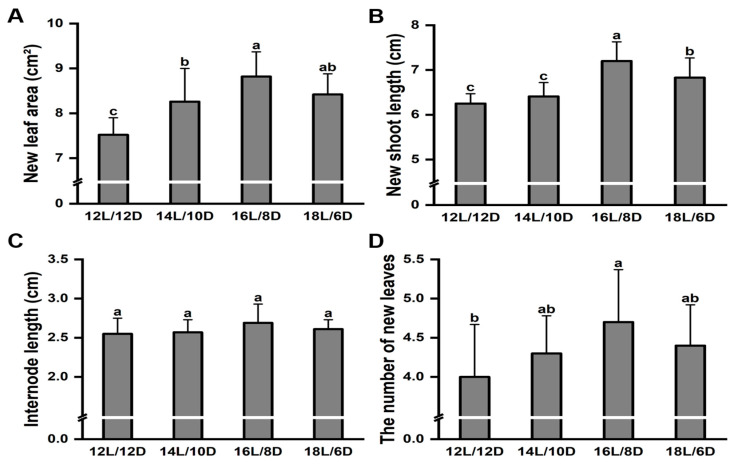
Effect of different photoperiod treatments on shoot growth in ‘Huangkui’. Effect of different photoperiod treatments on new leaf area (**A**), new shoot length (**B**), length of the internode (**C**), and the number of newly expanded leaves (**D**) of ‘Huangkui’. Note: Different lowercase letters on the top of the bar chart indicate significant differences at the level of *p* < 0.05.

To further compare the growth difference under different light treatments, the new leaf area, new shoot length, length of the internode, and the number of newly expanded leaves were analyzed. The greatest new leaf area (Figure 3A), new shoot length (Figure 3B), and number of newly expanded leaves (Figure 3D) were observed under the 16L/8D treatment. However, no significant difference was found in the length of the internode under different light treatments (Figure 3C).

### 2.5. Tea Plant’s Phenological Periods under Different Light Quality Treatments

**Figure 4:**
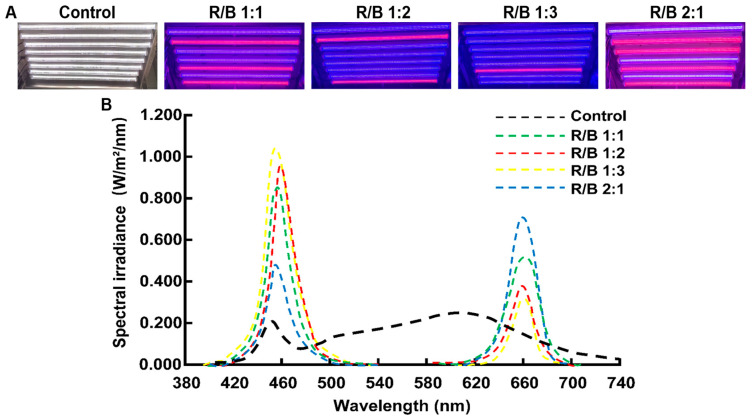
Spectral distribution of different light treatments. (**A**) Different light quality equipment, (**B**) Spectral composition in different light quality treatments.

The above results indicate that a 16L/8D photoperiod is a suitable treatment for the growth of ‘HuangKui’. To further explore the effect of different light qualities on tea growth under the 16L/8D photoperiod, the different lighting equipment is presented, and the ratios of red and blue light are shown in Figure 4. Significant differences in the growth of ‘HuangKui’ were found under different light-quality treatments.

**Figure 5:**
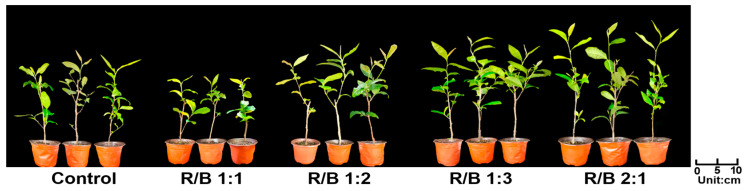
The effect of different light quality treatments on the growth of tea plants.

As shown in Figure 5, the R/B 2:1 treatment maximally promoted the growth of the tea plants. Additionally, the impact of different light quality treatments on the number of days of the tea plants’ phenological periods was investigated. ‘HuangKui’ germinated 3 days earlier under the R/B 2:1 treatment compared with the control. In contrast, the R/B 1:1 treatment impaired the growth of tea leaves, and similar results were found regarding the number of days until the appearance of one bud and one leaf, one bud and two leaves, and one bud and three leaves (Table 3).

### 2.6. Leaf Photosynthetic Performance under Different Light Quality Treatments

**Figure 6:**
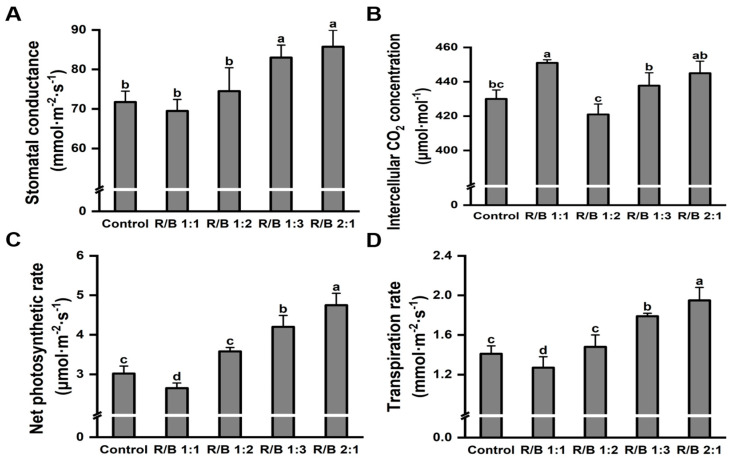
Effect of different light quality treatments on photosynthetic parameters of ‘Huangkui’. Effect of different light quality treatments on stomatal conductance (**A**), intercellular CO_2_ concentration (**B**), net photosynthetic rate (**C**), and transpiration rate (**D**) of ‘Huangkui’; Note: Different lowercase letters on the top of the bar chart indicate significant differences at the level of *p* < 0.05.

To further illustrate the effect of different light-quality treatments on the growth of the tea plants, the photosynthetic parameters were measured on the fully expanded second leaves of each treatment. Consistent with the above results, the highest level of stomatal conductance (Figure 6A) was detected under the R/B 2:1 treatment. Thus, the photosynthesis of ‘HuangKui’ was significantly higher by 57.28% under the R/B 2:1 treatment compared with the control (Figure 6C). Also, similar results were found in the intercellular CO_2_ concentration (Figure 6B) and transpiration rate (Figure 6D).

### 2.7. Growth of Tea Plants under Different Light Quality Treatments

**Figure 7:**
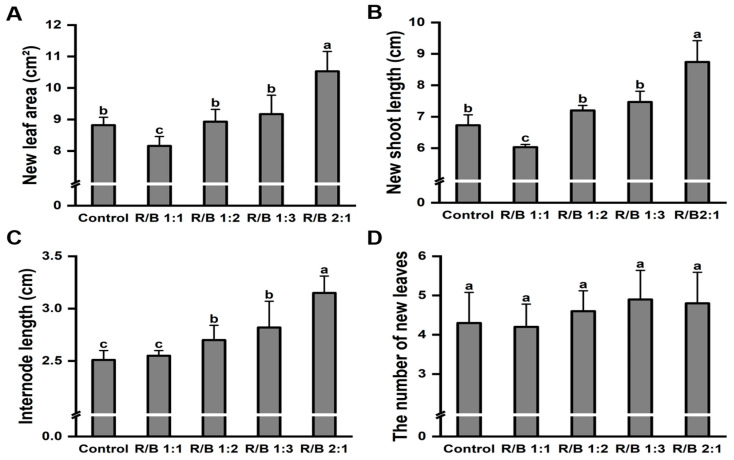
Effect of different light quality treatments on shoot growth in ‘Huangkui’. Effect of different light quality treatments on new leaf area (**A**), new shoot length (**B**), length of the internode (**C**), and the number of newly expanded leaves (**D**) of ‘Huangkui’. Note: Different lowercase letters on the top of the bar chart indicate significant differences at the level of *p* < 0.05.

Similarly, to further compare the growth difference under different light quality treatments, the new leaf area, new shoot length, length of the internode, and number of newly expanded leaves were measured. The highest new leaf area (Figure 7A), new shoot length (Figure 7B), and length of the internode (Figure 7C) were detected under the R/B 2:1 treatment compared with the control treatment, being significantly higher by 19.38%, 29.87%, and 25.50%, respectively. However, no significant difference was found in the number of newly expanded leaves under the different light-quality treatments (Figure 7D). Additionally, the R/B 1:1 treatment significantly impaired tea plant growth, and the new shoot length was significantly lower by 10.41%.

### 2.8. Tea Plant Quality under Different Light Quality Treatments

**Figure 8:**
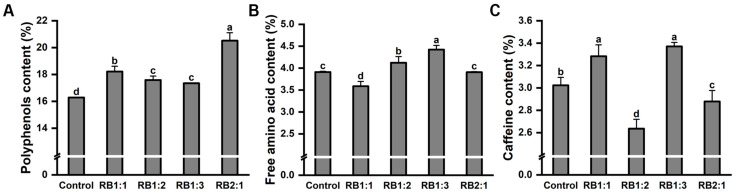
The effect of different light quality treatments on the quality in ‘Huangkui’. The effect of different light-quality treatments on polyphenol content (**A**), free amino acids (**B**), and caffeine content (**C**). Note: Different lowercase letters on the top of the bar chart indicate significant differences at the level of *p* < 0.05.

The above results indicate that R/B 2:1 is a suitable light-quality treatment for the germination and growth of ‘HuangKui’. To further investigate the effects of different light-quality treatments on tea quality, the tea polyphenol content, total free amino acid content, and caffeine content were analyzed using the first leaf from the top (Figure 8).

Among the different treatments, the R/B 2:1 treatment resulted in the highest level of tea polyphenols for ‘HuangKui’ (Figure 8A). Also, the levels of C (87.50%), EGCG (5.24%), GCG (105.52%), ECG (25.70%), and TC (5.83%) significantly increased under the R/B 2:1 treatment compared with those in the control treatment (Table 4). In addition, a higher ratio of blue light (R/B 1:2 and R/B 1:3) significantly increased the content of free amino acids by 5.45% and 13.12%, and no significant difference was found in the total free amino acid content under the R/B 2:1 treatment compared with the control. However, ‘HuangKui’ tea leaves under the R/B 1:1 treatment exhibited the lowest level of total free amino acids (Figure 8B). Moreover, compared with the control treatment, the contents of caffeine under the R/B 1:1 and R/B 1:3 treatments significantly increased by 8.60% and 11.52%, respectively. However, the R/B 1:2 and R/B 2:1 treatments exhibited a decrease in the caffeine content compared with the control treatment (Figure 8C).

### 2.9. The Hormone Content and Gene Expression Levels under Different Light Qualities

**Figure 9:**
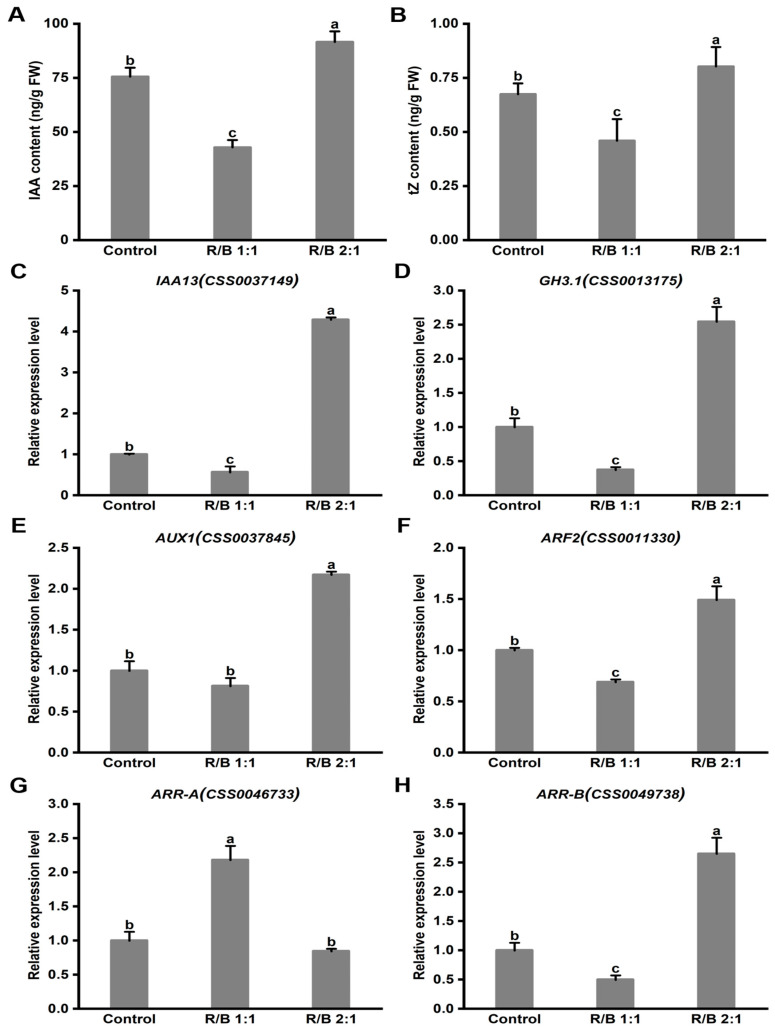
The hormone content of IAA (**A**) and tZ (**B**) and expression levels of *CsIAA13* (**C**), *CsGH3.1* (**D**), *CsAUX1* (**E**), *CsARF2* (**F**), *CsARR-A* (**G**) and *CsARR-B* (**H**) in ‘Huangkui’ leaves grown under R/B 1:1, R/B 2:1 and control treatment. Note: Different lowercase letters on the top of the bar chart indicate significant differences at the level of *p* < 0.05.

As mentioned in the introduction, auxin and cytokinin play an important role in plant growth. Thus, the auxin (IAA) and trans-zeatin (tZ) contents in ‘HuangKui’ under different light quality treatments were detected. The results indicate that tea plants with earlier germination (R/B 2:1) exhibited higher contents of IAA and tZ (Figure 9A). However, lower accumulation levels of IAA and tZ were detected in tea plants that germinated later (R/B 1:1) (Figure 9B).

To further clarify the promotion mechanism, the expression level of the auxin- and cytokinin-related genes in the first leaf from the top was analyzed via *qRT-PCR*.

The results show that the auxin-related gene expression levels of *CsIAA13*, *CsGH3.1*, *CsAUX1*, and *CsARF2* in the R/B 2:1 treatment were significantly higher than those in the control (Figure 9C–F). In addition, a significantly lower expression level of *CsARR-A* (negative regulator) was detected under the R/B 2:1 treatment than the control, while the opposite result was found for *CsARR-B* (positive regulator) (Figure 9G,H).

## 3. Discussion

The timing of bud flush is one of the most important agronomic traits of tea plants. Tea cultivars with an early timing of bud flush are highly valued for economic development. However, under natural conditions, the late timing of bud flush in the albino tea plant cultivar ‘HuangKui’ limits this tea’s economic value. Light serves as the energy source for plant photosynthesis, influencing plant growth and development. Plants perceive different wavelengths of light through various photoreceptors that respond to different regions of the absorption spectrum [19], playing an important role in the timing of bud flush of various plants [20,21].

The results of our study reveal that the appropriate condition to promote the bud flush of ‘HuangKui’ was the 16L/8D photoperiod (Table 1). Similar results reported that a 16L/8D photoperiod promoted earlier budburst in two co-occurring Mediterranean oaks (*Quercus ilex* L. subsp. *Ballota* and *Q. faginea* Lam.) [22]. Generally, a longer photoperiod contributes to an increase in light capture and canopy photosynthesis, thus increasing the chlorophyll content and germination [23]. However, excessively long photoperiods can cause an overabundance of light energy, which disrupts the photosynthetic chloroplasts, thereby adversely affecting plant growth and development [24]. Therefore, in our study, a lower total chlorophyll content was detected under the 18L/6D treatment compared with the 16L/8D treatment (Table 2). It may be that the longer photoperiods led to excess light energy, which impeded photosynthetic chloroplast metabolism and tea plant growth. Also, Šrajer Gajdošik reported that the most favorable growth conditions for industrial hemp (*Cannabis sativa* L. subsp. *sativa*) were observed under a 16L/8D photoperiod compared with those under a 20L/4D photoperiod [25]. Additionally, another previous study reported that excessively long photoperiods lead to the appearance and development of photo-oxidation or photodamage [26]. Therefore, our results imply that a 16L/8D photoperiod is an appropriate means of timing bud flush for the albino tea variety ‘HuangKui’, while the longer photoperiod (18L/6D) hindered chlorophyll biosynthesis and photosynthetic ability.

The blue light band (400–500 nm) and red light band (600–700 nm) are important components associated with the sunlight spectrum, playing a primary role in plant growth and development [27]. It is well known that red light wavelengths perfectly fit the absorption peak of chlorophylls and phytochromes, and thus, a high ratio of red light promotes plant growth [28]. In our study, a high ratio of red light (R/B = 2:1) significantly promoted tea plant growth compared with low ratios of red light (R/B = 1:1, 1:2, and 1:3). Similar results reported that a ratio of red/blue light of 2:1 was the most suitable light source for the growth of *Haematococcus pluvialis*, producing a higher biomass and photosynthetic pigment content than a red/blue light 1:2 treatment [29]. Also, the maximum growth rate of duckweed (*Lemna minor* L.) (11.37 g/m^2^/day) was observed at a red/blue ratio of 2:1 compared with a red/blue ratio of 1:4 [30]. Consistent with the above results, our results show that a ratio of red/blue light of 2:1 significantly promoted tea plant growth under a 16L/8D photoperiod (Table 3).

As mentioned above, the R/B 2:1 treatment significantly promoted the growth of tea plants under a 16L/8D photoperiod [31]. Plants perceive light signals via multiple sensory photoreceptors. Additionally, light signaling coordinates with plant hormones such as auxins and cytokinins in regulating plant germination and growth [32,33]. As is well known, auxin is one of the plant hormones associated with cell elongation. The auxin family candidates *GH3*, *Aux/IAA*, and *SAUR* play a significant role in plant growth [34,35]. In addition, cytokinins are widely recognized as key regulators in promoting germination [36].

Therefore, to further reveal the promotion mechanism, the related plant hormone contents of leaves were detected (Figure 9A,B). Our results show that ‘HuangKui’ under the R/B 2:1 treatment exhibited a higher content of auxin (IAA) compared with those under the R/B 1:1 and control treatments under the 16L/8D photoperiod (Figure 9A). To further establish a correlation between the levels of transcripts of the studied genes and IAA level, *GH3.1* [37,38], *IAA13* [39], *ARF2* [40], and *AUX1* [41], which affect IAA signaling pathways, were analyzed. Our results show that higher auxin-related gene expression levels of *CsGH3.1*, *CsIAA13*, *CsARF2*, and *CsAUX1* were detected under R/B 2:1 with earlier germination (Figure 9C–F). Previous studies also reported the role of auxin-related genes in IAA synthesis and plant growth. For example, the addition of 0.05 μM 1-naphthylacetic acid to a medium of Rhododendron cells significantly increased the expression level of *DUHoIAA13*, resulting in a higher IAA content, thereby effectively completing the regeneration of buds from leaves in vitro [42]. Also, *SlARF2*-overexpressing tomato (*Solanum lycopersicum*) displayed significantly increased IAA contents, which enhanced tomato lateral root formation [43]. Additionally, the overexpression of *OsAUX1* in rice led to an increase in the IAA content, resulting in an increased rate of lateral root initiation in rice plants in response to local nitrate supply [44]. Consistent with the above study, our study indicated that a higher ratio of red light (R/B 2:1) induced a higher expression level of auxin-related genes (*CsGH3.1*, *CsIAA13*, *CsARF2*, and *CsAUX1*) and significantly increased the IAA content, thus promoting the germination of tea plants.

Moreover, cytokinins are widely recognized as key regulators in promoting germination [36]. Two response regulators (*ARR-A* and *ARR-B*) are involved in cytokinin signal transduction, and they play opposite roles in the germination of plant buds [45]. In our study, the highest trans-zeatin (tZ) content was found under the R/B 2:1 treatment (Figure 9B), and the cytokinin-related gene expression level of *CsARR-B* under R/B 2:1 was significantly higher than that in the control (Figure 9H). In Arabidopsis, *ARR-B* positively promotes bud regeneration by directly activating *WUS* expression [46]. In addition, *BcARR-B* positively regulated cytokinin synthesis in cabbage (*Brassica campestris* L.) [47]. In contrast, the lowest cytokinin-related gene expression level of *CsARR-A* was found under the R/B 2:1 treatment (Figure 9G). The overexpression of *AtARR-A* in Arabidopsis decreased the trans-zeatin (tZ) content and resulted in a significant reduction in hypocotyl elongation [48]. Therefore, in our study, the higher expression level of *ARR-B* and the lower expression level of *ARR-A* regulated cytokinin synthesis, thus promoting the germination and growth of ‘HuangKui’.

Therefore, our results show that R/B 2:1 under a 16L/8D photoperiod is an appropriate means of timing the bud flush for the albino tea variety ‘HuangKui’, which may be due to the specific plant hormone signal transduction. However, the interaction between hormones and their specific molecular mechanisms still needs to be further investigated.

## 4. Materials and Methods

### 4.1. Plant Material and Experimental Settings

The plants of the albino tea variety ‘HuangKui’ (2-year-old seedlings) were obtained from Langxi County, Xuancheng City, Anhui Province, China. The tea plants were carefully washed and planted in a plastic pot with a radius of 4 cm and a height of 10 cm that was filled with peat, perlite, and vermiculite (2:1:1). Then, the tea plants were transferred to an artificial climate chamber (Anhui Agricultural University, located at 31°520′ N 117°150′ E). The conditions were as follows: a temperature of around 25 °C and a humidity between 70% and 75%. The tea plants were grown for 12 days before the light treatments were imposed. The LEDs were equipped with light plates (Hangzhou Linan Jiayu Technology Co., Ltd., Hangzhou, China). The photosynthetic photon flux density (PPFD) in the climate chamber was set to 200 μmol·m^−2^ s^−1^ irradiance for all plants.

The light treatments with different photoperiods included 12L/12D, 14L/10D, 16L/8D, and 18L/6D, and the different light treatments were simulated from the solar spectrum. The light treatments with different red/blue (R/B) light ratios included R/B 1:1, R/B 1:2, R/B 1:3, and R/B 2:1. The light distribution was recorded using a SPIC-300AW (Hangzhou Yuanfang Optoelectronic Information Co., Ltd., Hangzhou, China) spectrometer, as shown in Table 5.

### 4.2. Determination of Phenological Period Timing

The timing of the budburst, one bud and one leaf, one bud and two leaves, and one bud and three leaves, was assessed visually. These visual assessments were performed using the method described by Yi et al. [49]. This method is recognized for the identification of phenological periods in tea plants.

### 4.3. Determination of Photosynthetic Characteristics

The photosynthetic parameters of fully expanded second leaves from twelve plants in each treatment group were recorded using a portable photosynthesizer (CIRAS-3, PP Inc., Amesbury, MA, USA), as described in Yang et al. [50]. During the measurements, the light intensity was maintained at a constant level of 200 μmol m^−2^ s^−1^, the temperature was strictly controlled within the range of 25 ± 1 °C, and the CO_2_ concentration was strictly controlled at 400 ± 10 μmol mol^−1^.

### 4.4. Chlorophyll Concentration

The first and second leaves of tea plants were collected. The leaves were weighed and ground with 10 milliliters of 80% acetone. The liquid supernatants were filtered, and the samples were centrifuged at 4000× *g* for 10 min. The concentrations of chlorophyll a (Chl a) and chlorophyll b (Chl b) were measured using a spectrophotometer at 663 nm (OD (optical density)_663_) and 646 nm (OD_646_). The contents of chlorophyll a and b were calculated using the following formulas:

CChl.a (mg g^−1^ FW) = (12.21 × OD_663_ − 2.81 × OD_646_)/(1000 × FW) × V

CChl.b (mg g^−1^ FW) = (20.13 × OD_646_ − 5.03 × OD_663_)/(1000 × FW) × V

where V and FW indicate the volume of the reaction system and the weight of the isolated tea leaves, respectively [51].

### 4.5. Determination of Tea Plant Growth

For the growth analysis, the growth indicators were measured once new buds had ceased to sprout (*n* = 12). The lengths of new shoots and internodes were accurately measured by using a vernier caliper (DL91150, Delix Group, Ninghai County, Ningbo City, China), and the area of the leaves was measured by using a specialized area meter (AM-350, ADC Bioscience UK Ltd., Oxfordshire, UK).

### 4.6. Tea Polyphenols, Free Amino Acids, and Caffeine

The concentrations of tea polyphenols, free amino acids, caffeine, and catechin components were determined in fresh leaves (second leaves) collected from tea plants. Tea polyphenols were determined using an ultraviolet (UV) spectrophotometer (UV T5, Shanghai, China) at 765 nm, followed by the Folin–Ciocalteu method [52]. The determination of free amino acids was based on the ninhydrin coloration staining method [53]. In addition, the extraction and detection methods of catechins were based on high-performance liquid chromatography (HPLC) analysis following the protocol by Ye et al. [54].

### 4.7. Hormone Determination

Hormone extraction and analysis were conducted as described by Kijidani et al. [55] and Zeng et al. [56]. Briefly, hormones were extracted from 300 mg of powdered tea leaves (fresh weight) and transferred to 15 mL screw-cap tubes. Then, 3000 μL of a 2-propanol/water/concentrated HCl solution (2:1:0.002, *v*/*v*) was initially added as the solvent. Subsequently, the samples were agitated in a shaker at 4 °C for 30 min. Then, 6000 μL of dichloromethane was added, and the mixture was agitated further for 30 min at 4 °C. Centrifugation at 4000 r/min for 10 min resulted in the formation of two distinct layers, with plant debris positioned at the interface. The lower layer (1 mL) was concentrated with a nitrogen evaporator for 1 h, redissolved in 1 mL of methyl alcohol, vortexed, centrifuged at 4000 r/min for 10 min at 4 °C, and analyzed by liquid chromatography/mass spectrometry (LC/MS).

IAA (97% content) and tZ (98% content) as internal standards, and plant hormone standard solutions were prepared in methanol/water/formic acid (50:50:0.1%, *v*/*v*). For IAA analysis, an ACQUITY UPLC BEH C18 column (2.1 mm × 100 mm, 1.7 μm, Waters, Milford, MA, USA) was used with a mobile phase of acetonitrile and distilled water, both containing 0.1% (*v*/*v*) formic acid, at a flow rate of 0.3 mL/min. In addition, for tZ analysis, a CORTECS UPLC T3 column (2.1 mm × 100 mm, 1.6 μm, Waters, Milford, MA, USA) was used with a mobile phase consisting of methanol and distilled water, both containing 0.1% (*v*/*v*) formic acid, at a flow rate of 0.3 mL/min. Quantitative analyses were performed based on the calibration curves of the standards.

### 4.8. Primer Design and Quantitative Real-Time Polymerase Chain Reaction (PCR) (qRT-PCR)

qRT–PCR and RNA extraction were performed following the method presented by Wang et al. [57]. In our experiments, *CsGAPDH* was selected as a reference control, and primers for the validated genes were designed using Primer 3 Plus. Relative expression levels were calculated using the 2^−ΔΔCt^ method [58]. All of the primers used in this study are shown in Table S1.

### 4.9. Statistical Analysis

All measurements of different replicate samples were analyzed. All data analyses were performed using SPSS 26.0 software. Data are presented as the mean ± standard deviation (SD). The statistical analysis included a one-way analysis of variance (ANOVA), and statistical significance was calculated using Duncan’s multiple range test at a significance level of *p* < 0.05. 

## 5. Conclusions

In this study, we found that the R/B 2:1 treatment under a 16L/8D photoperiod was an appropriate mode for promoting the germination and growth of the albino tea cultivar ‘HuangKui’.

## Figures and Tables

**Table 1:** The effect of different photoperiod treatments on the tea plant phenological periods.

Photoperiod Treatments	Budding	One Bud and One Leaf	One Bud and Two Leaves	One Bud and Three Leaves
	Days	Days	Days	Days
12L/12D	10 d	16 d	21 d	27 d
14L/10D	9 d	14 d	19 d	25 d
16L/8D	8 d	12 d	17 d	24 d
18L/6D	8 d	13 d	18 d	25 d

**Table 2:** The effects of different photoperiod treatments on chlorophyll content in ‘HuangKui’. Means followed by different letters differ significantly by Duncan (*p* < 0.05).

Photoperiod Treatments	Chlorophyll A (mg/g FW)	Chlorophyll B (mg/g FW)	Chlorophyll (A + B) (mg/g FW)
12L/12D	0.563 ± 0.011 b	0.136 ± 0.020 b	0.699 ± 0.013 d
14L/10D	0.575 ± 0.004 b	0.159 ± 0.009 ab	0.734 ± 0.011 c
16L/8D	0.649 ± 0.022 a	0.189 ± 0.034 a	0.838 ± 0.013 a
18L/6D	0.642 ± 0.015 a	0.142 ± 0.009 b	0.784 ± 0.010 b

**Table 3:** The impact of different light quality treatments on the formation days of tea plant phenological periods.

Light Quality Treatments	Budding	One Bud and One Leaf	One Bud and Two Leaves	One Bud and Three Leaves
	Days	Days	Days	Days
Control	8 d	12 d	17 d	24 d
R/B 1:1	10 d	14 d	17 d	26 d
R/B 1:2	7 d	12 d	16 d	22 d
R/B 1:3	6 d	10 d	15 d	21 d
R/B 2:1	5 d	9 d	14 d	18 d

**Table 4:** The effect of light quality treatments on the catechins acid in ‘HuangKui’. Means followed by different letters differ significantly by Duncan (*p* < 0.05).

Light Quality Treatments	Control	R/B 1:1	R/B 1:2	R/B 1:3	R/B 2:1
GC	5.16 ± 0.21 a	3.99 ± 0.40 b	3.60 ± 0.43 b	3.43 ± 0.78 b	3.68 ± 0.48 b
EGC	34.47 ± 0.42 ab	33.22 ± 1.64 bc	32.20 ± 0.23 c	29.86 ± 0.39 d	35.00 ± 0.31 a
C	0.96 ± 0.02 c	0.95 ± 0.00 c	1.78 ± 0.06 b	2.21 ± 0.04 a	1.80 ± 0.01 b
EC	13.85 ± 0.10 ab	13.37 ± 0.54 b	12.00 ± 0.13 c	12.35 ± 0.36 c	14.40 ± 0.30 a
EGCG	66.18 ± 0.18 b	57.83 ± 0.37 e	64.73 ± 0.87 c	62.86 ± 0.49 d	69.65 ± 1.39 a
GCG	1.45 ± 0.40 c	2.38 ± 0.28 b	2.47 ± 0.04 b	3.06 ± 0.03 a	2.98 ± 0.05 a
ECG	8.56 ± 0.46 bc	10.52 ± 0.36 a	9.16 ± 0.10 b	8.28 ± 0.29 c	10.76 ± 0.55 a
TC	130.64 ± 0.74 b	122.27 ± 0.62 d	125.92 ± 0.85 c	122.06 ± 1.20 d	138.26 ± 2.15 a

Note: GC, (−)-gallocatechin; EGC, (−)-epigallocatechin; C, (+)-catechin; EC (+)-epicatechin; EGCG, (−)-epigallocatechin gallate; GCG, (−)-gallocatechin gallate; ECG, (−)-epicatechin gallate; TC, total catechins.

**Table 5:** The ratio of the emission spectral distribution of the LED light sources.

Light Quality Treatments	Control	R/B 1:1	R/B 1:2	R/B 1:3	R/B 2:1
Number of R/B LED Tube Assemblies	/	4/2	3/3	3/2	3/2
Half-width HW/nm	170.6	22.5	22.5	22.7	24.6
Irradiance Ee (W/m^2^)	53.31	42.0	33.8	36.1	34.4
Light intensity range μmol^−1^ m^−2^ s^−1^	201~223	192~229	204~247	193~233	207~238
Red light band share (R)	LED plant full spectrum light	50%	33%	25%	66%
Lue light band share (B)	LED plant full spectrum light	50%	66%	75%	33%

## Data Availability

The data presented in this study are available on request from the corresponding author. The data are not publicly available due to privacy.

## Supplementary Materials

The following supporting information can be downloaded at: https://www.mdpi.com/article/10.3390/plants13131782/s1, Table S1: Primers used in this study.
