# Supplementary material for: Effects of Photoperiod and Light Quality on Germination and Growth of Camellia sinensis ‘HuangKui’
Source: Plants (Basel). 2024 Jun 27;13(13):1782. doi: 10.3390/plants13131782 (PMC11244327; doi:10.3390/plants13131782)
Supplement: Supplementary file 1 [file plants-13-01782-s001.zip › plants-3068367-supplementary.pdf]

Table S1. Primers used in this study

Gene name	Gene ID	Forward primer sequences (5'-3')	Reverse primer sequences (5'-3')
CsARR-B	CSS0049738	CCAAATGCAATCGAGCGGAG	ATGCCTCAATTCGAACCGGT
CsARR-A	CSS0046733	CAGGTGTTTGGAGGAAGGGG	GACGAAAGAGGCGATGGTGA
CsGH3.1	CSS0013175	AGTGAGCACTTCAAGACCCG	TGAGTTGGAGGAAGCGGATG
CsIAA13	CSS0037149	GAGCTGGAGTTGGGTCTTGG	ACCTCTGGGGGAGCAACATA
CsAUX1	CSS0037845	GGTGAGATTGCCTGTGGTCA	GAAGTTTGTCATGCTCGCCC
CsARF2	CSS0011330	TCCTGAACCAAAGTGCTCCA	GGCGTTCCCTCTTTCGATCA
